# Interconnection of bacterial and phytoplanktonic communities with hydrochemical parameters from ice and under-ice water in coastal zone of Lake Baikal

**DOI:** 10.1038/s41598-020-66519-3

**Published:** 2020-07-06

**Authors:** Yu. S. Bukin, N. A. Bondarenko, I. I. Rusanov, N. V. Pimenov, S. V. Bukin, T. V. Pogodaeva, S. M. Chernitsyna, O. V. Shubenkova, V. G. Ivanov, A. S. Zakharenko, T. I. Zemskaya

**Affiliations:** 1 Limnological Institute SB RAS, Irkutsk, 664033 Russia; 2 Winogradsky Institute of Microbiology, Federal Research Centre “Fundamentals of Biotechnology” RAS, Moscow, 117312 Russia; 3 Irkutsk State University, Irkutsk, 664003 Russia

**Keywords:** Ecosystem ecology, Freshwater ecology, Systems analysis

## Abstract

We analysed the relationship between the chemical complex (concentration of dissolved ions, nutrients, pH) and biological parameters (primary production, biomass of phytoplankton, abundance and activity of bacterial communities) at estuaries of rivers and coastal waters of Southern Baikal during the under-ice period. Correlation network analysis revealed CO_2_ to be the main limiting factor for the development of algae and microbial communities in the coastal zone of Lake Baikal. This study indicates that primarily reverse synthesis of bicarbonate and carbonate ions associated with the development of phytoplankton and accumulation of dissolved CO_2_ during photosynthesis regulates pH in the Baikal water. We did not detect the anthropogenic factors that influence the change in pH and acidification. Near the Listvyanka settlement (Lake Baikal, Listvennichnaya Bay), there was a great number of organotrophs and thermotolerant bacteria with low bacterioplankton activity and high concentration of organic carbon. This evidences eutrophication due to the influx of organic matter having an anthropogenic source. Nutrients produced during the bacterial destruction of this matter may explain the changes in bottom phytocenoses of Listvennichnaya Bay.

## Introduction

Human activity causes a wide range of stresses of various scales in ecosystems^1^. Many scientific studies have assessed the impact of many of these stresses on aquatic ecosystems, which led to important basic knowledge in the field of limnology and ecology. Eutrophication and acidification^2^ are considered to be the main processes determining trophic changes in many freshwater ecosystems. Recently, there is a growing understanding of the need to determine the combined effects of multiple factors^3–5^, since the widespread occurrence of diverse stresses requires an accompanying assessment of multiple loads for individual ecosystems. In addition, in recent years, a consistent reaction of lakes to global climate changes has been demonstrated in different regions of the world, which emphasizes the need to study the role of both regional and local factors in regulating the response of individual lakes to this impact^6^. Comprehensive research in this area involving methods of ecology, hydrochemistry and hydrology is important for developing environmental standards to ensure the achievement of local water purity targets and the proper functioning of ecosystems.

Lake Baikal hosts an extremely complex ecosystem with a plethora of interspecies interactions^7^. The purity of its waters is passively supported by the low mineralization of the river inflow and actively maintained by the orchestrated work of the algal community (phytoplankton) and bacterioplankton in the pelagic zone^8–10^. The complex biochemical processes happening in this community maintain the balance of dissolved inorganic and organic matter in the water of Lake Baikal. Phytoplankton development leads to the accumulation of dissolved salts that contain biogenic elements as well as the transformation of those salts into insoluble forms. Bacterioplankton, in turn, is responsible for the destruction of organic matter produced by the algae. In this way, dissolved matter is constantly leaving the water column and sinking to the bottom of the lake floor where it forms sediments. Diatom algae, which build their cell walls from dissolved silica, are a key component of sub-ice phytoplankton (a primary producer in lake Baikal)^7,8^. Thus, the stability of microalgal and bacterial communities maintains the water quality in Lake Baikal^7–9^.

During the last few years, there have been significant changes in the lake ecosystem, particularly in coastal zones^11–13^. In some low-depth areas (i.e., littoral zone), previously dominant species of the bottom phytocenoses have been replaced. Spirogyra, a filamentous alga indicative of eutrophication, has become widespread^11–14^. Studies have shown that minor ecological changes are also happening in the pelagic zone^15,16^. In particular, diatom algae endemic to the pelagic zone are being replaced by cosmopolitan species; this leads to a decrease in the total primary production of the lake^15,16^. Additionally, an increasing abundance of cyanobacteria has been reported in Lake Baikal’s benthic communities^16^. It was hypothesized that changes in coastal ecosystems were triggered by an influx of biogenic elements, and that this influx, in turn, was caused by tourist activity near the lake compounded by a lack of sewage treatment facilities^11–13,17^. In contrast, changes in pelagic ecosystems are attributable to global climate change, which has shortened ice periods and increased the average temperature of surface waters^15,16^.

The phytoplanktonic and bacterioplanktonic communities of Lake Baikal are adapted to a specific combination of environmental factors^18–20^ such as total dissolved salts, biogenic element availability, and water pH. Carbon is introduced to the lake ecosystem primarily via the photosynthetic carbon dioxide assimilation^21–23^, and in small quantities during bacterial consumption of methane and other hydrocarbons coming from the lake bottom^24^. Carbon dioxide concentration is a major factor affecting water pH due to the the reversible synthesis of hydrocarbonate (HCO_3_^−^) and carbonate (CO_3_^−^) ions^25^. An increase in the anthropogenic load on the lake ecosystem can also affect water pH. In Lake Baikal, acidification may happen in coastal areas near the outlets of heavily polluted rivers. Acidification is occurring in the rivers of Lake Baikal’s southern basin^26,27^, caused by the emission of sulfur and nitrogen oxides from industry and heat-electric generation plants^27^. The pelagic zone in Lake Baikal’s center remains relatively buffered from pH changes because of its volume; therefore, it can be used as a reference point when studying the lake’s coastal areas.

Intensive phytoplankton growth during the spring accounts for most of Lake Baikal’s primary production^19,20,28–30^. These massive blooms happen in the euphotic zone (0–25 m) because of the accumulation of biogenic elements under the ice, including phosphates, nitrates, and carbon dioxide, as well as the dissolved silica necessary for diatom algae growth. During the spring (late March to early April for the southern basin), under-ice waters are heated by increasing solar radiation, and the massive diatom bloom begins, peaking near the end of ice season. This algal bloom produces easily-oxidized organic matter that allows bacterioplankton growth to intensify. Some phytoplankton and bacterioplankton is consumed by zooplankton, and organic matter moves to higher trophic layers. With the increase in solar radiation, algae and bacteria also begin developing on the ice-water boundary and in the ice pores, forming so-called ice communities^31^.

The main goal of this work was to study the hydrochemical parameters of water and the functional characteristics of the communities of algae and bacteria in ice water and in ice samples in estuarine zones of rivers with varying degrees of anthropogenic load, and assess the impact of stress factors of eutrophication and acidification on the course of key environmental processes. The complex analysis was aimed at studying the mutual influence of hydrochemical parameters, biological parameters, and the pH of the lake littoral.

## Results

### Sampling area and investigated parameters

**Figure 1:**
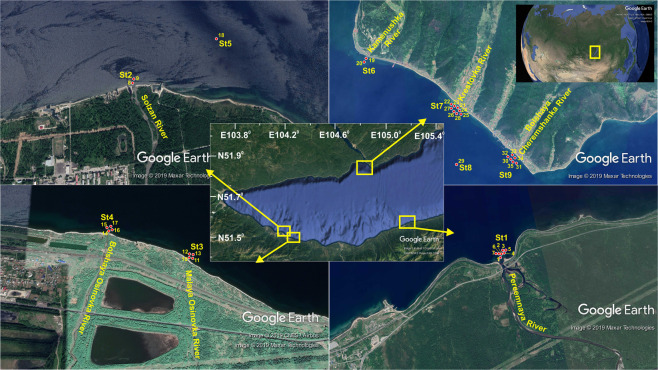
Map of the sampling sites. Sample numbers correspond to the numbers in in the table with the initial data (https://github.com/barnsys/bac_phyt_communities). Geographic coordinates of stations (degrees and decimal places): St1 51.56891 N, 105.16609; St2 51.49722 N, 104.15836 E; St3 51.50056 N, 104.25354 E; St4 51.50056 N, 104.24403 E; St5 51.53875 N, 104.19746 E; St6 51.84457 N, 104.87505 E; St7 51.85535 N, 104.85970 E; St8 51.86710 N, 104.83247 E; St9 51.84429 N, 104.83949 E. Maps were taken from the *Google Earth Pro* software (https://www.google.com/intl/ru/earth/versions/#earth-pro). «Inkscape» (https://inkscape.org/) free graphics editor was used to edit images and apply additional marks to maps.

In March 2018, water, ice, and snow were sampled from Lake Baikal’s southeastern and southwestern coasts in the river mouths and from the lake waters near the rivers. Samples were also taken from two reference stations in the lake’s pelagic zone (51.53875 N, 104.19746 E and 51.86710 N, 104.83247 E) (Fig. 1). The lower reaches of the Solzan (51.49722 N, 104.15836 E), Bolshaya Osinovka (51.50056 N, 104.24403 E) and Malaya Osinovka (51.50056 N, 104.25354 E) rivers pass through the town of Baikalsk and its industrial area. The Pereemnaya River (51.56891 N, 105.16609 E) does not pass through any settlements in its entire course. The lower reaches of the Kamenushka (51.84457 N, 104.87505 E), Krestovka (51.85535 N, 104.85970 E), and Bolshaya Cheremshanaya rivers (51.84429 N, 104.83949 E) pass through the Listvyanka settlement, which exhibits heavy tourism activity. The water from these rivers enters the Listvennichny Bay, which has fast water exchange with Lake Baikal.

This study examined two parameter categories. The first category describes the biological processes that occur in the studied waters (biological parameters): concentrations of organic carbon (C_org_) and chlorophyll *a* (Chl_a), total phytoplankton mass (∑PB), total rate of methane oxidation (∑MO), count of organotrophic bacteria (OB), count of thermotolerant bacteria cultivated at 22 °C (TMC22C), count of thermotolerant bacteria cultivated at 37 °C (TMC37C), biogenic oxygen depletion (BOD), and total primary production rate (∑PP), which is the rate of light-dependent CO_2_ assimilation, total bacterial count (NB), and total bacterial primary production (∑BP) which is the rate of dark*-*assimilation CO_2_. The second category includes the hydrochemical parameters relevant for the microbial community: concentrations of Na^+^, K^+^, Ca^2+^, Mg^2+^, HCO_3_^−^, Cl^−^; concentrations of biogenic elements such as N(NO_2_^−^), N(NO_3_^−^), S(SO_4_^−^), N(NH_4_^+^), P(PO_4_^3−^); concentration of dissolved carbon in carbon dioxide C(CO_2_) and methane C(CH_4_); oxygen concentration (O_2_); chemical oxygen depletion (COD); and water pH. Table with biological and hydrochemical parameters values are available at: https://github.com/barnsys/bac_phyt_communities.

### Statistical analysis of hydrochemical and biological parameters of samples from ice and under-ice water

**Figure 2:**
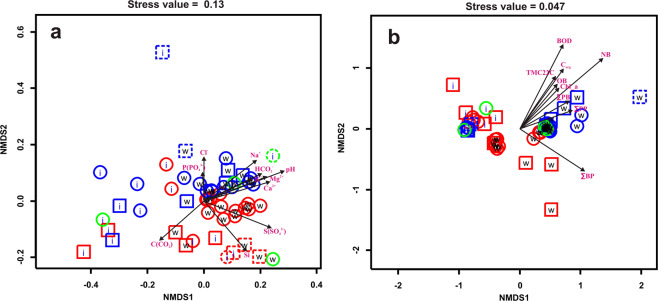
Scatter plots of sampling sites in two-dimensional NMDS space. (**a**) parameters describing biological processes are treated as explained variables, hydrochemical parameters are treated as explanatory ones, (**b**) hydrochemical parameters are explained, biological parameters are explanatory. Red — eastern coast, blue — western coast; green circles represent pelagic samples; circles — samples taken from the lake near river mouths, squares — river mouths; dashed blue squares are samples taken near the mouth of Bolshaya Cheremshanka river; dashed red pointers are samples taken near Bolshaya Osinovka and Malaya Osinovka rivers; w — water samples, i — ice samples. Plots show only the vectors of gradient for explanatory variables significantly affecting the datasets of explained variables (Tables 1 and 2).

The result of the analysis of the relationship of hydrochemical and biological parameters is shown on the NMDS scatter plots Fig. 2. The statistical support for each factors are shown in Tables 1 and 2.

**Table 1:** PERMANOVA analysis results for the assessing the statistical significance of the influence of hydrochemical parameters in sampling sites on the complex of biological parameters.

Parameter name	Average value	Coefficient of variation (CV)	PERMANOVA analysis	
			R^2^	P value
**Quantitative variables**				
Na^+^ concentration (mg L^−1^)	2.16	0.77	***0.30***	***0.0009***
K^+^ concentration (mg L^−1^)	0.75	0.69	***0.24***	***0.0009***
Ca^2+^ concentration (mg L^−1^)	10.09	0.66	***0.27***	***0.0009***
Mg^2+^ concentration (mg L^−1^)	1.88	0.75	***0.26***	***0.0009***
HCO_3_^−^ concentration (mg L^−1^)	37.99	0.75	***0.27***	***0.0009***
C(CO_2_) concentration (mg L^−1^)	1.29	1.55	***0.08***	***0.038***
Cl^-^ concentration (mg L^−1^)	0.64	1.31	***0.27***	***0.0009***
N(NO_2_^−^) concentration (mg L^−1^)	0.0030	2.44	0.05	0.09
N(NO_3_^−^) concentration (mg L^−1^)	0.30	1.98	0.05	0.09
S(SO_4_^2−^) concentration (mg L^−1^)	2.027	0.77	***0.06***	***0.027***
N(NH_4_^+^) concentration (mg L^−1^)	0.012	1.86	***0.18***	***0.003***
P(PO4_3_^−^) concentration (mg L^−1^)	0.013	2.26	***0.30***	***0.0009***
Si concentration (mg L^−1^)	0.86	1.13	***0.07***	***0.019***
Chemical oxygen depletion (COD) (mg O_2_ L^−1^)	3.25	0.64		
pH	7.36	0.10	***0.245***	***0.0009***
**Qualitative variables**				
Sampling site			***0.27***	***0.015***
Sampling zone(river mouth, near-mouth littoral water, pelagic water)			0.04	0.3
Coast(western, eastern)			***0.09***	***0.038***
Biotope (water or ice sample)			***0.171***	***0.0009***

**Table 2:** PERMANOVA analysis results for the assessing the statistical significance of the influence of the biological parameters in sampling sites on the complex of hydrochemical parameters.

Parameter name	Average value	Coefficient of variation (CV)	PERMANOVA analysis	
			R^2^	P value
**Quantitative variables**				
Organic carbon concentration (C_org_) (mg L^−1^)	1.94	0.4	***0.177***	***0.0009***
Total phytoplankton biomass (∑PB) (μg L^−1^)	48.08	0.9	***0.165***	***0.0009***
Chlorophyll a concentration (Сhl_a) (μg L^−1^)	0.96	1.23	***0.144***	***0.0009***
Total rate of primary production (∑PP) (μg C L^−1^ day^−1^)	7.68	1.28	***0.154***	***0.0009***
Biological oxygen depletion (BOD) (mg O_2_ L^−1^)	0.71	0.77	***0.23***	***0.0009***
Total bacterial count (NB)(cells mL^−1^)	930.58	1.07	***0.44***	***0.0009***
Count of organotrophic bacteria (OB) (CFU mL^−1^)	390.54	3.20	***0.12***	***0.0009***
Count of thermotolerant bacteria cultivating at 22 °C (TMC22C)(CFU mL^−1^)	219.78	4.5	***0.12***	***0.0019***
Count of thermotolerant bacteria cultivating at 37 °С (TMC37C)(CFU mL^−1^)	17.14	3.36	0.024	0.30
Total bacterial primary production (∑BP) (μg C L^−1^ day^−1^)	0.63	0.66	***0.28***	***0.0009***
**Qualitative variables**				
Sampling site			0.24	0.06
Sampling zone(river mouth, near-mouth littoral water, pelagic water)			0.04	0.34
Coast(western, eastern)			***0.11***	***0.021***
Biotope (water or ice sample)			***0.46***	***0.0009***

When the biological parameters were treated as dependent variables, the following qualitative and quantitative factors were found to significantly affect them: concentrations of Na^+^, K^+^, Ca^2+^, Mg^2+^, C(CO_2_), Cl^−^, S(SO_4_^2−^), P(PO4_3_^−^), Si, HCO_3_^−^; pH; the coast of Lake Baikal (western or eastern); and the biotope (water column or ice) (PERMANOVA analysis Table 1). The strongest effects (R^2^ > 0.2) were observed for concentrations of Na^+^, K^+^, Ca^2+^, Mg^2+^, Cl^−^, HCO_3_^−^, P(PO4_3_^−^), pH and sampling site (Table 1). Two major divisions between samples can be observed on the NMDS scatter plot (Fig. 2a). The first is more pronounced and separates the ice and water column samples. This division is statistically supported by the PERMANOVA analysis, since the biotope (ice or water column) is a significant factor that influences the values of biological parameters (Table 1). Water column featured higher concentrations of Na^+^, Ca^2+^, Mg^2+^, S(SO_4_^2−^), and HCO_3_^−^, higher pH and lower C(CO_2_) concentration. The second is less pronounced and separates the samples from the eastern and western coasts of Lake Baikal. This separation also confirms by PERMANOVA analysis (Table 1). Western samples as a rule had higher Cl^−^ and P(PO4_3_^−^) concentration as well as lower concentrations of S(SO_4_^2^)^−^ and silicon (Si). A particularly high concentration of Cl^−^ and P(PO4_3_^−^) was in the sample of water and ice from the mouth of the Cheremshanka river (Fig. 2a).

When hydrochemical parameters were treated as dependent variables (Fig. 2b, PERMANOVA analysis Table 2), the following qualitative and quantitative factors were found to significantly affect them: C_org_, ∑PB, Сhl_a, ∑PP, BOD, NB, ∑BP, OB, TMC22C, biotope (water or ice) and Coast (western, eastern). BOD, NB, ∑BP, and biotope had the strongest effects (R^2^ > 0.2). The ice and water samples (Fig. 2b, PERMANOVA analysis Table 2) differ in chemical composition. This usually comes from increased biological activity in the water samples (i.e., higher values of C_org_, ∑PB, Сhl_a, ∑PP, BOD, NB, OB and TMC22C). Some western water samples (Fig. 2b) exhibited very high values of biological parameters C_org_, ∑PB, Сhl_a, ∑PP, BOD, NB, ∑BP, OB and TMC22C. Particularly high values of these parameters were observed for the water samples from the mouth of Bolshaya Cheremshanka river.

The investigated area of the western coast of Lake Baikal in the area of Listvyanka settlement, especially in the mouths of the rivers, also showed high values of biological parameters (implying high rates of biological processes) (Fig. 2). In addition to biological parameters that significantly affect hydrochemical variables, this sample had the highest counts of organotrophic bacteria (OB), thermotolerant bacteria cultivated at 22 °C (TMC22C), and thermotolerant bacteria cultivated at 37 °C (TMC37C). These samples showed high concentrations of Cl^−^, which is an indicator of anthropogenic pollution. One of the ice samples taken at the same site had an even higher Cl^−^ concentration and lower bacterial and phytoplankton activity (roughly similar to other ice samples from the lake) (Fig. 2). All rivers (Kamenushka, Krestovka, and Bolshaya Cheremshanaya) flowing into the lake in the area pass through the Listvyanka settlement in sections ranging from 1.6 to 2.8 km (Fig. 1).

For both hydrochemical and biological parameters, parameter variance did not appear to correlate with the significance of the parameter’s effect on the diversity of sampling sites (Tables 1 and 2). Parameters with high coefficient of variation between the samples (CV > 1) may have little impact on the patterns of other parameters. In contrast, parameters with low coefficient of variation (CV < 1) may affect other parameters with high significance and R^2^ (R^2^ > 0.3).

### Analysis of the correlation between hydrochemical and biological parameters of samples from under-ice water

**Figure 3:**
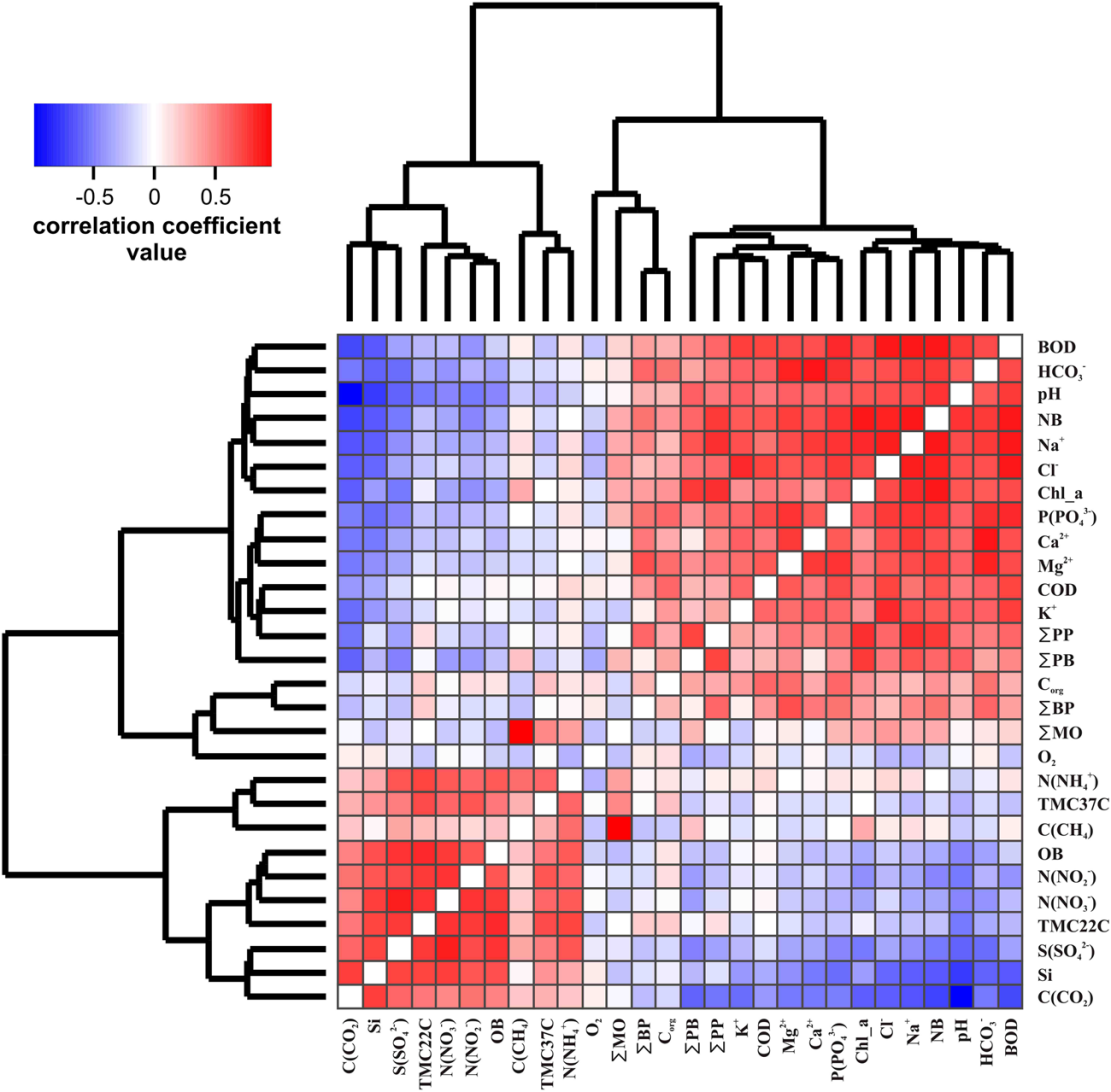
Heat map of pairwise correlation coefficients for biological and hydrochemical parameters. Parameters in the heat map are ordered according to the clustering based on similarity of correlation coefficient vectors. Clusters discussed in text (A, B, C, D) are shown on the clustering dendrogram.

Because the water samples were significantly different from the ice samples on both hydrochemical and biological parameters, only the water samples were used in the correlation analysis and heat map; the number of ice samples (*n* = 16) was not sufficient for a separate multivariate correlation analysis. Results of the multivariate correlation analysis are shown in Fig. 3 as a heat map. Based on the clustering, several groups of intercorrelated variables were identified. The largest group (cluster A) included hydrochemical parameters such as the P(PO_4_^3−^), Mg^2+^, Na^+^, K^+^, Ca^2+^, HCO_3_^−^, Cl^−^; COD. This group also included biological parameters such as C_org_, Сhl_a, ∑PB, ∑PP, NB, ∑BP, and BOD. All of these parameters showed positive pairwise correlations with each other. The second group (cluster B) included three variables: N(NH_4_^+^), C(CH_4_), and the count of thermotolerant bacteria cultivated at 37 °C (TMC37C). These three variables showed slightly positive pairwise correlations. The third group (cluster C) included O_2_ concentration and ∑MO. In most cases, these two parameters were weakly correlated, whether to each other or to other variables. The only exception was the correlation between ∑MO and C(CH_4_). The correlation value for this pair was close to 1. The last group (cluster D) included seven parameters: N(NO_2_^−^), N(NO_3_^−^), S(SO_4_^2^), C(CO_2_), Si, TMC22C, and OB. All variables within this cluster were strongly positively correlated with each other.

When examining the areas of the heat map describing inter-cluster correlation coefficients, all values were either close to zero or negative. There were two pronounced groups with high positive correlation coefficients for intragroup correlations as well as high negative correlation coefficients for intergroup relationships (closely connected groups). The first group included a part of cluster A, particularly such hydrochemical parameters as P(PO_4_^3−^), Mg^2+^, Cl^−^, HCO_3_^−^, Na^+^, Ca^2+^, K^+^, and water pH as well as such biological parameters as ∑PB, Сhl_a, NB, and BOD. The second group consisted of all variables from cluster D (i.e., N(NO_2_^−^), N(NO_3_^−^), S(SO_4_^2−^), C(CO_2_), Si, TMC22C, and OB). In general, an increase in the parameters of the former group caused a decrease in the parameters of the latter, and vice versa.

### Network analysis of the correlation relationships between hydrochemical and biological parameters of samples from under-ice water

**Figure 4:**
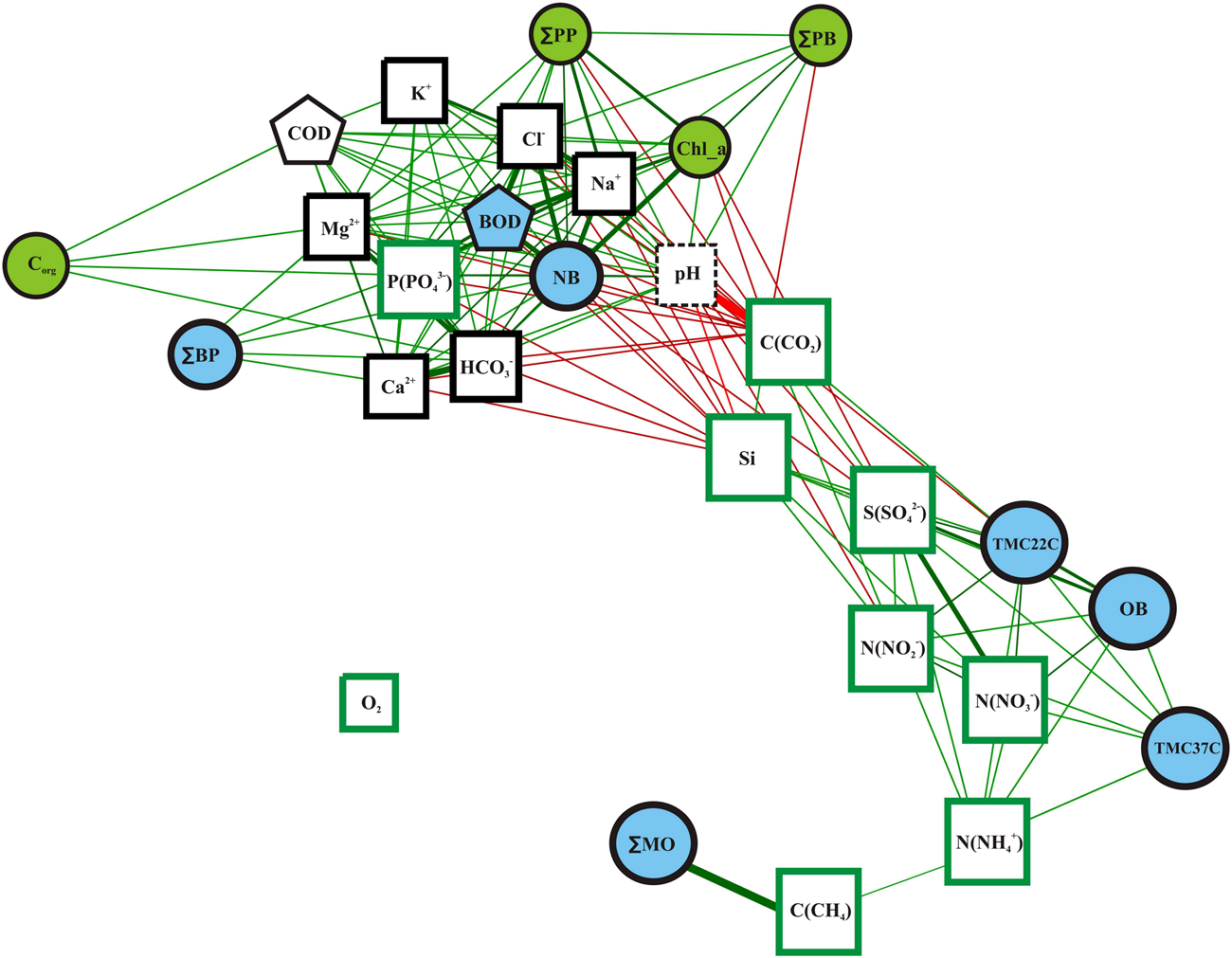
The network of correlations between all measured parameters (both biological and hydrochemical). Blue shapes mark the parameters related to the bacterial activity, green circles mark the parameters related to phytoplankton activity, black squares — mineral ion concentrations, green squares — concentrations of biogenic elements, pentagons mark the rate of biogenic and chemical oxidation of organic matter. Green edges mean significant positive correlations, red edges mean significant negative correlations.

After correcting the P values for pairwise correlations between biological and hydrochemical parameters, only correlation coefficients (*r*) with absolute values above 0.5 were significant (p < 0.05). These pairwise correlation coefficients were used to build the interaction network (Fig. 4).

As with the heat map, there were two major groups of parameters in the interaction network. The first group included hydrochemical parameters such as P(PO_4_^3−^)^−^, Mg^2+^, Na^+^, K^+^, Ca^2+^, HCO_3_^−^, Cl^−^, pH, and COD; and biological parameters such as C_org,_ Сhl_a, ∑PB, ∑PP, ∑BP, and BOD. All of these parameters were positively correlated to each other. The second group included N(NO_2_^−^), N(NO_3_^−^), S(SO_4_^2−^), C(CO_2_), P(PO_4_^3−^), Si, С(CH_4_), OB, TMC22C, TMC37C, and ∑MO. All of these were also positively correlated to each other. In contrast, all of the edges between these two groups corresponded to negative correlations. In other words, increasing any parameter or group of parameters from the first group will (through a chain of interactions) lead to an increase in all parameters from the first group as well as a decrease in all parameters from the second group. The same can be said about the parameters in the second group. Thus, the results of the interaction network analysis are comparable to the results of the heat map.

Based on the number of edges, the parameters were separated into three groups. The first group included the parameters with a lot of connections (11–18). More specifically, this group included P(PO_4_^3−^), Mg^2+^, Na^+^, K^+^, Ca^2+^, HCO_3_^−^, Cl^−^, S(SO_4_^2−^), Si, C(CO_2_), COD, BOD, NB, Сhl_a, ∑PB, ∑PP, and water pH. Among these parameters, S(SO_4_^2−^), pH, Si, and C(CO_2_) had the highest number of negative correlations. The second group had a moderate number of correlations (6–10 edges). Among its members are N(NH_4_^+^), N(NO_2_^−^), N(NO_3_^−^), ∑PB, OB, TMC22C, and TMC37C. Parameters in this group had very few negative correlations (0 or 1). The third group featured the outliers with 1–4 connections: C(CH_4_), C_org_, and ∑MO. Oxygen concentration (O_2_) had no significant correlations at r > = 0.5.

All nodes had relatively low betweenness centrality (ranging from 0 to 0.174, with the highest possible value being 1) (Table 3). Dissolved carbon dioxide concentration (C(CO_2_)) had the highest betweenness centrality value. This means that the shortest path between any two nodes in the network often passes through the C(CO_2_) node. C(CO_2_) also had the highest number of negative correlations, as shown by the number of negative correlation edges in the network. The nodes with the next highest betweenness centrality values were S(SO_4_^2−^), N(NH_4_^+^), and N(NO_2_^−^) at the values of 0.105, 0.137, and 0.134, respectively. The two nitrogen concentrations N(NH_4_^+^) and N(NO_2_^−^) did not have a notable number of connections (7 and 9 edges, respectively). The group with betweenness centrality values between 0.05 and 0.1 included C(CH_4_), Si, and water pH. Si^−^ and water pH had a high number of correlations with neighboring nodes, while C(CH_4_) was positively correlated with only two other parameters.

**Table 3:** Numeric parameters of biological and hydrochemical variables as the nodes in correlation network built for water samples.

Parameter name	Betweenness centrality	Total number of correlation edges	Number of positive correlations	Number of negative correlations
Na^+^ concentration	0	15	13	2
K^+^ concentration	0	11	10	1
Ca^2+^ concentration	0.028	13	11	2
Mg^2+^ concentration	0.026	15	14	1
HCO_3_^−^ concentration	0.017	16	13	3
C(CO_2_) concentration	0.174	17	4	13
C(CH_4_) concentration	0.071	2	2	0
Cl^−^ concentration	0.011	15	13	2
N(NO_2_^−^) concentration	0.134	9	8	1
N(NO_3_^−^) concentration	0	7	7	0
S(SO_4_^2−^) concentration	0.105	12	8	4
N(NH_4_^+^) concentration	0.137	7	7	0
P(PO_4_^3−^) concentration	0.031	16	14	2
Si concentration	0.057	14	6	8
O_2_ concentration	0	0	0	0
Chemical oxygen depletion (COD)	0.017	12	12	0
pH	0.066	18	13	5
Organic carbon concentration (C_org_)	0	4	4	0
Chlorophyll a concentration (Сhl_a)	0	7	6	1
Total phytoplankton biomass (∑PB)	0.023	13	11	2
Total rate of primary production (∑PP)	0.009	11	10	1
Biological oxygen depletion (BOD)	0	14	12	2
Total bacterial count (NB)	0.028	17	14	3
Count of organotrophic bacteria (OB)	0.003	7	7	0
Count of thermotolerant bacteria cultivating at 22 °C (TMC22C)	0.006	9	8	1
Count of thermotolerant bacteria cultivating at 37 °С (TMC37C)	0	6	6	0
Total rate of methane oxidation (∑MO)	0	1	1	0
Total bacterial primary production (∑BP)	0	6	6	0

## Discussion

The results of our study show that the coastal waters of Lake Baikal near Listvyanka settlement are under heavy anthropogenic load. All the Baikal tributaries in this area, as well as its coastal waters and ice, had an increased concentration of Cl^−^ anion (from 1 to 4 mg L^−1^ in near Listvyanka compared to 0.11–0.6 mg L^−1^ at other sampling points), a marker of anthropogenic pollution^32^. Previous research has reported increased concentrations of Cl^−^ in this area; those studies similarly interpreted this as evidence of anthropogenic load^13,17^. This raises questions regarding the exact nature of the pollutants. Water samples from this area also had high concentrations of organic carbon (from 2.5 to 4.85 mg L^−1^ in near Listvyanka compared to 0.85–1.99 mg L^−1^ at other sampling points) and high rates of biological oxygen depletion (from 1.6 to 3.2 mg O_2_ L^−1^ in near Listvyanka compared to 0.1–1.2 mg O_2_ L^−1^ at other sampling points). In addition, counts of organotrophic bacteria (from 550 to 6800 CFU mL^−1^ in near Listvyanka compared to 1–490 CFU mL^−1^ at other sampling points) and thermotolerant organotrophic bacteria (from 400 to 6000 CFU mL^−1^ TMC22C in near Listvyanka compared to 1–100 CFU mL^−1^ TMC22C at other sampling points) were significantly increased. Thus, it can be concluded that tributary rivers in this area are responsible for bringing organic matter from residential waste water into Lake Baikal. This waste water is not processed owing to the absence of central canalization and sewage treatment facilities in the settlement. This conclusion is further supported by the composition of these rivers’ phytoplankton, which come mostly from the Cryptomonas genus, a common inhabitant of retention ponds. It should also be noted that we did not detect any significant increase in the concentrations of dissolved biogenic elements compared to water samples from other areas. At the same time, the littoral near Listvyanka settlement is one of the sites where we observed a massive development of filamentous algae and changes in bottom phytocenoses^11–13,33^. These changes may reflect a scenario in which residential waste, including a large volume of fecal mass, is washed into rivers by precipitation and then flows into Lake Baikal’s littoral areas. A part of this organic matter sinks to the bottom, where it is destroyed by organotrophic bacteria. It is known^34^ that human fecal matter contains increased concentrations of organic carbon, nitrogen, and phosphorus, which can be oxidized into bioavailable carbon dioxide, nitrates, and phosphates. As a result, near-bottom waters suffer a local increase in the concentrations of biogenic elements. Thus, in the littoral environment of lake Baikal near Listvyanka settlement at depths of 2–15 m, an abundance of biogenic elements, high water transparency (and therefore high solar radiation near the bottom), and an absence of strong waves combine to create a favorable environment for filamentous algae like Spirogyra. This scenario filamentous algae development in Lake Baikal at depths usually free of such species is similar to that observed in the Great Lakes^35^. The lack of a noticeable increase in the concentrations of biogenic elements in water column can be explained by the high rate of water exchange between Listvennichny Bay and the pelagic waters of Lake Baikal.

On the eastern coast of Lake Baikal, in both river mouths and surrounding areas, we observed an increase in the count of organotrophic bacteria. However, this increase was not as critical as on the western coast near Listvennichny Bay. Although the lower reaches of the studied rivers (Solzan, Malaya Osinovka, Bolshaya Osinovka) pass through the town of Baikalsk, Cl^−^ concentration was not increased in these samples; moreover, the concentration of organic carbon and the biological oxidation rate were similar or even lower than at the reference sites in the pelagic area. This can be explained by the fact that Baikalsk has both centralized sewers and sewage treatment facilities where easily oxidizable organic matter is destroyed by bacteria, while organic nitrogen and phosphorus are converted into their soluble forms. In addition, the Baikalsk discharge pipe opens at a depth of 40 m, which is below the euphotic layer. This speeds up the mixing of wastewater with the main body of Baikalian water. One of the tributary rivers, the Pereemnaya, has not settlements at all. As such, the anthropogenic load on the Pereemnaya River is lowered. In all of the samples from the eastern coast, we observed increased concentrations of dissolved silicon and sulfates. This is primarily explained by the geological composition of the area that these rivers flow through; however, these results could also be affected by the fact that these rivers bring a much larger volume of water into Lake Baikal than do the rivers on the western coast^35^. The significant increase in sulfate concentration in the mouths of both Osinovka rivers is related to the fact that they flow near the Baikalsk Pulp and Paper Mill’s storage area for liquid waste, and this liquid waste contains a substantial amount of dissolved sulfates^36^.

Compared to the under-ice water, the ice samples had decreased concentrations of Na^+^, K^+^, Ca^2+^, and Mg^2+^. This likely occurs because mineral ions are frozen out of the liquid phase during ice formation. Only one ice sample, taken from a reference station near the eastern coast of Lake Baikal, had an increased pH. This increased pH is attributable to the phytoplankton community that had begun to develop in the ice and that community’s consumption of carbon dioxide. In general, the increased biological activity of ice communities and the increased mineralization of ice samples from the eastern coast are explained by environmental factors related to local winters. More specifically, there is a substantial buildup of ice caused by the inflow of silicon- and biogen-rich water from the rivers.

Our study shows that phytoplankton development in the under-ice community of Lake Baikal has remained largely unchanged in recent decades^7,8,20,25,28,29^. As solar radiation and temperature increase, pronounced algal development starts in the biogen-rich water. When the supply of biogenic elements is depleted, vegetation wanes. Our study fits with the results of earlier research reporting a decrease in carbon dioxide concentration during phytoplankton development^21–23^. In our dataset, carbon dioxide concentration ranked second after pH in terms of the number of correlations with other parameters. It exhibited more correlations than N(NO_2_^−^), N(NO_3_^−^), S(SO_4_^2−^), N(NH_4_^+^), P(PO_4_^3−^), Si, and O_2_ (Table 3). In addition, carbon dioxide concentration had the highest betweenness centrality in the interaction network (Table 3) and was significantly correlated with the total biological activity of both phytoplankton and bacterioplankton (Table 2). Moreover, an analysis of the data from^22^ shows that during spring, the concentration of carbon dioxide decreased faster as a percentage of the initial value than the concentration of other nutrients. It can be hypothesized that dissolved carbon dioxide is the major regulator of the primary production rate in Lake Baikal’s under-ice phytoplankton. As phytoplankton grow and the supply of carbon dioxide is depleted, pH significantly increases. In these conditions, plankton is suppressed not only by the shortage of carbon available for biosynthesis but also by the inhibition of the proton pump apparatus responsible for the active import of other nutrient elements into algal cells.

A series of studies estimating the effect that oxidative stress caused by atmospheric pollution with sulfur dioxide (SO_2_) has on freshwater lake phytoplankton detected the suppression photosynthesis, a decrease in primary production, and a lowering of microalgae taxonomic diversity at pH values under 5.7^37–42^. The lowest pH value observed in our work was 5.76 in the ice sample taken near the Pereemnaya River on the eastern coast of Lake Baikal. This sample had a low rate of primary production and insignificant algal biomass. The concentration of carbon dioxide in this sample was higher than in all other samples, while the sulfate concentration was lower. These data confirm that a high concentration of CO_2_ was responsible for low pH at this site. Thus, we can conclude that the water of Lake Baikal does not undergo significant acidification that could affect the activity of phytoplankton and bacterioplankton under the ice. Just as before, water pH is predominantly regulated by the photosynthetic activity of phytoplankton, which shifts the equilibrium in the reversible reactions of hydrocarbonate and carbonate synthesis. It is important to note that pH was significantly negatively correlated with the concentration of both nitrates N(NO_3_^−^) and sulfates S(SO_4_^2−^). These strong acidic residues lower pH with increasing their concentration and are actively accumulated by phytoplankton. Thus, it is possible that this mechanism contributes to the changing pH balance in Baikalian waters. The role that photosynthetic N(NO_3_^−^) and S(SO_4_^−^) accumulation have on pH regulation merits additional study. Notably, however, study^43^ provide evidence for a connection between sulfurous compounds and the activity of under-ice phytoplankton.

It is important to note that in Lake Baikal’s littoral area, chlorophyll concentration and phytoplankton biomass were positively correlated with pH, which varied from 5.76 to 8.16. In other freshwater lakes and rivers, primary phytoplankton production was negatively correlated with pH values in this range^37^. Thus, the Baikalian phytoplankton complex is adapted to function under low concentrations of carbon dioxide. Microalgae are able to consume CO_2_ and other biogenic elements at high pH.

The concentrations of all biogenic elements in the water, including (N(NO_2_^−^), N(NO_3_^−^), S(SO_4_^2−^), N(NH_4_^+^), Si, and C(CO_2_)), were directly or indirectly negatively correlated with biological activity. These correlations are attributable to the consumption of these elements by the growing phytoplankton (Figs. 3 and 4). The only exception to this rule was phosphate (P(PO_4_^−^)) concentration. Phosphate concentration was positively correlated with the biological parameters. We hypothesize that phosphorus is a catalyst for all biological processes: the higher its concentration, the quicker phytoplankton grow. It should also be noted that the concentration of dissolved phosphates was extremely low in most samples, and very nearly at the lower limit of quantitative measurement. The role that phosphates play in phytoplankton development requires additional study.

Finally, the structure of the bacterial community was complex. Organotrophic and thermotolerant bacteria, which oxidize easily mineralizable organic matter, fell into a cluster of variables positively correlated with the biogenic element concentrations (N(NO_2_^−^), N(NO_3_^−^), S(SO_4_^2−^), N(NH_4_^+^), Si) which are formed from organic compounds consumed by these bacteria. The total bacterial count as measured by the fluorescent method (NB variable) (Figs. 3 and 4), including the count of cyanobacteria, fell into the same cluster as the other primary producers. Like diatoms, cyanobacteria consume biogenic elements and produce organic matter. Some other bacteria from NB are probably forming algae-bacterial communities and are responsible for the destruction of the specific forms of phytoplankton organic matter^44,45^. This would explain the close correlation between NB and biogenic oxidation rate (BOD) (Figs. 3 and 4). Unsurprisingly, the activity of methanotrophic bacteria was correlated to dissolved methane concentration (Figs. 3 and 4).

Our work is one of the first to apply an integrated approach to studying the mechanisms of functioning of the ecosystem of the water column of Lake Baikal. It allows us to conclude that when developing the standards for acceptable anthropogenic load on oligotrophic and ultra-oligotrophic lakes, it is necessary to take into account not only pollution by dissolved biogenic elements N(NO_2_^−^), N(NO_3_^−^), S(SO_4_^2−^), N(NH_4_^+^), P(PO_4_^3−^), but also the flow of organic carbon. Large flow of organic matter of anthropogenic origin under conditions of high oxygen concentration will activate organotrophic bacteria producing carbon dioxide (CO_2_). Large concentrations of carbon dioxide will activate photosynthesis processes causing undesirable consequences, including mass developments of filamentous algae and cyanobacteria.

## Materials and Methods

### Sampling

Water was sampled with a Niskin bottle at all sites, including from the river mouth as well as from the lake at a distance of 50–100 m in various directions from the mouth. Ice and snow were sampled at the same sites. At every point for chemical, microbiological and phytoplankton analyses, samples were taken in triplicate, followed by separate studys and averaging of the result. In the analyses of the primary production rate and total bacterial primary production rate, samples were taken in duplicate, followed by separate studys and averaging of the result.

### Chemical analysis

Chemical analyses were performed using the methods commonly accepted in fresh water hydrochemistry^46–48^. Cations (Na^+^, K^+^, Ca_2_^+^, Mg_2_^+^) were detected using atomic absorption and flame emission methods (relative precision 2–3%). Anions (Cl^−^, SO4^2−^, HCO_3_^−^, NO_3_^−^) were measured using HPLC (relative precision 5–10%). Biogenic element concentrations were measured using colorymetry (relative precision of −1,5% for phosphates and −3–5% for nitrates). Ammonium nitrogen was measured using the indofenol method (relative precision up to 5%). The reliability of the measured biogenic element concentrations was supported by a quality control analysis conducted according to EANET guidelines for testing reference samples of surface water. The measured concentrations of major ions were also controlled by calculating the error in ion balance and comparing the estimated and measured values of specific electrical conductivity. Dissolved oxygen was measured using iodometry according to Winkler, with a relative error of 1%^49^ at the sampling site. The chemical oxygen depletion (COD) of oxidizing organic compounds was measured using the permanganate index. Biogenic oxygen depletion (BOD) was estimated with bichromate oxidizability. The relative error of both methods did not reach more than 10%. The concentration of organic carbon (C_org_) was estimated using the catalytic high-temperature oxidation of samples at 850 °С followed by measurement of the produced СО_2_ with an infrared detector on Vario TOC cube high-temperature carbon analyzer (Germany). The final result was calculated from the average of three measurements (standard deviation under 0.01%).

Methane concentration was measured with the «Headspace» method^50^ on the «Эхо_EW» gas chromatograph with a flame ionization detector (Novosibirsk, Russia).

The pH of water, melted snow, and melted ice core samples was measured with a WTW pH 3310 device at the sampling site.

CO_2_ concentrations were calculated based on HCO_3_^−^ concentration, pH, and the total mineral content of the solution^22^.

Concentrations of NO_2_^−^, NO_3_^−^, SO_4_^2−^, NH_4_^+^, PO_4_^3−^, and CO_2_ were converted into the concentrations of corresponding biogenic elements, N(NO_2_^−^), N(NO_3_^−^), S(SO_4_^2−^), N(NH_4_^+^), P(PO_4_^3−^), C(CO_2_).

### Microbiological analysis

Microbiological analyses of water, ice, and snow samples were performed according to the МУК 4.2.1018-01 and МУК 4.2.1884-04. The total counts of colony-forming mesophilic aerobic and facultative anaerobic bacteria were estimated by culturing on meat-and-peptone agar at 37 °С for 24 h (TMC37C) and at 22 °С for 72 h (TMC22C), respectively. To estimate the count of organotrophic bacteria we used a medium based on fish-and-peptone agar (РПА:10)^51^. Petri dishes with the samples were incubated at 22 °С for 7 days, after which the colonies were counted. To measure total bacterial count (NB), the water, melted ice, and melted snow samples were fixed with 4% formaldehyde, stained with DAPI fluorochrome^52^, passed through polycarbonate filters (25 mm diameter, 0.2 μm pore size; Isopore, Merck, Germany) by means of a manual vacuum pump and analyzed under an epifluorescent microscope (AxioImager.M1, Carl Zeiss).

### Total photosynthetic primary production rate (∑PP) and total bacterial primary production rate (∑BP)

Sampling for the analysis and estimation of primary production rates was performed as described before. In accordance with previously published protocols, measurements of photosynthesis intensity and bacterial assimilation of carbon dioxide were taken using the radiocarbon method^53,54^.

### Microbial methane oxidation (MO)

In accordance with previously published protocols, MO in sub-ice water samples was measured with the radiocarbon method (^14^СН_4_)^55^.

### Phytoplankton identification

For phytoplankton identification, we fixed 1 L of water with Lugol’s solution and then concentrated the phytoplankton via sedimentation. Algae were counted twice in a 0.1 mL Nageotte chamber under Peraval light microscopes with ×720 and ×1200 magnification. Algal biomass was calculated from the algal number using individual cell volumes^56^. To determine biovolume, 100–200 cells of each species were measured.

The chlorophyll *a* content was determined using the standard spectrophotometric method^57^.

### Statistical analysis of the data

All numeric data were assembled into a single table; its rows correspond to the sampling sites, and its columns correspond to the measured parameters. Missing data were replaced with the averages for a given parameter^58^. Oxygen concentration (O_2_), dissolved methane (C(CH_4_)), and total rate of methane oxidation (∑MO) were excluded from multidimensional scaling because technical problems prevented the estimation of these parameters for ice probes; moreover, using average values for all ice samples would have biased the data.

The overall ordination of the sampling sites on all parameters was examined using non-metric multidimensional scaling (NMDS). Multidimensional scaling and analysis of significant explanatory variables were performed in R, using the «vegan»^59^ package based on the tutorial^60^.

All data were transformed to eliminate the physical dimensions by ranging from zero to 1. For NMDS, the distance matrix was calculated using the Euclidean distance metric. Two analyses were performed. In the first analysis, biological parameters were treated as the dependent variables, while the abiotic hydrochemical factors were treated the explanatory variables. In the second analysis, calculations were reversed; the hydrochemical parameters were treated as the dependent variables, while the biological parameters were treated as the explanatory variables. This approach allowed us to characterize the interactions between two groups of variables in both directions. Fitting of the gradient vectors of quantitative explanatory variables in NMDS scatter plot was done using the «vegan» package function^60^.

Based on the Euclidean distance matrix, a PERMANOVA^61^ analysis in «vegan» package was also performed to determine the relationship between dependent and explanatory variables. The analysis gives the values of R^2^ coefficients which defined proportion of the variance in the dependent variables that is predictable from the independent variables. The reliability of the value of R^2^ was calculated using 1000 permutations.

Pairwise correlations between all parameters (either biological or hydrochemical) were estimated with Spearman’s *r* correlation coefficient. Pairwise correlations were visualized with a heat map generated using «gplots» in R. Lines and columns in the correlation matrix were clustered and grouped in order of similarity (i.e., Euclidean distance metric and the complete-link clustering method).

In addition, the correlation matrix was visualized as a network using the «qgraph» and «igraph» packages in R. The network topology was based on the number of links and correlation coefficients between neighboring nodes. The more links the nodes form among themselves, the closer they were placed in the network. Only reliable correlation coefficients (*p* < 0.05) were included in the network. P values for the correlation coefficients were calculated using Spearman’s «W» statistics and corrected for false discovery rate in multiple comparisons using the Benjamini–Hochberg equation. To estimate the overall connectedness of the nodes in this network, we used normalized betweenness centrality^62^ and each node’s edge count.

## Data Availability

Scripts for R programming language used for statistic analysis and initial data table with biological and hydrochemical parameters values are available at: https://github.com/barnsys/bac_phyt_communities.
